# The effect of non-thermal atmospheric plasma on the production and activity of recombinant phytase enzyme

**DOI:** 10.1038/s41598-018-34239-4

**Published:** 2018-11-09

**Authors:** Mahsa Farasat, Sareh Arjmand, Seyed Omid Ranaei Siadat, Yahya Sefidbakht, Hamid Ghomi

**Affiliations:** 1 Laser and Plasma research Institute, Shahid Beheshti University, G. C., Tehran, Iran; 2 Protein Research Center, Shahid Beheshti University, G. C., Tehran, Iran

## Abstract

Atmospheric pressure cold plasma (ACP) is introduced as a useful tool in a variety of biological applications. Proteins are the most abundant macromolecules in living systems with a central role in all biological processes. These organic molecules are modified by ACP exposure that is responsible for many of ACP’s biological effects. This study evaluated the effect of ACP on the production of recombinant phytase in yeast *Pichia pastoris* (*P. pastoris*) as well as the structure and function of the phytase enzyme. The results indicated that yeast cells treated with ACP, directly or indirectly, produced higher amounts of recombinant phytase, which was associated with the time of ACP treatment. The exposure of commercial phytase solution with ACP caused a significant increase in the enzyme activity (125%) after 4 hours. Evaluation of the phytase solution by far- and near-UV circular dichroism (CD) and fluorescence analysis indicated that this protein maintained its secondary structure when exposed to ACP while the tertiary structure was slightly unfolded. The effects of heat and H_2_O_2_ on the phytase structure and function were compared with the effect of ACP treatment. The modification of Cys, Tyr and Trp amino acids upon reactive oxygen/nitrogen spices was simulated using a molecular dynamics approach. RMSF and RMSD analysis suggested that this structural alteration occurs owing to changes made by reactive species in accessible amino acids.

## Introduction

Plasma is described as the fourth state of matter. It is classified into thermal and non-thermal types. The thermal plasma is defined by a thermal equilibrium between ions and electrons while cold plasma shows a strong non-equilibrium of temperature between ions and electrons^1^. Initially, cold plasma was produced in a low-pressure condition. Developments in the field of plasma physics led to the production of ACP, which made it operationally more flexible. It also enabled it to generate reactive oxygen species (ROS) and reactive nitrogen species (RNS) at low temperatures. These features made ACP an extremely useful tool in biological and biomedical applications such as wound healing^2^, sterilization^3^, blood coagulation^4^, cancer therapy^5^, cell proliferation^6^, and living tissue treatment^7^. Although biological effects of plasma have been investigated by many researchers, the exact mechanisms of action have not been exactly specified so far. It seems that the biological effects of ACP are mostly related to its influence on the proteins as the functional and structural units of life, that comprise more than 50% of the cell dry weight. ACP can affect the protein’s structure and function by oxidizing the surface amino acids and making changes in the secondary and/or tertiary structures^8–11^. These changes may lead to positive or negative effects on the protein stability or activity, which is different by case.

In addition to its various biological effects, this feature of ACP makes it an important technique in some industries, as well. For instance, ACP can improve the activity of proteins that have industrial applications or inhibits undesirable enzymes in industrial processes. One of the widely used proteins in industrial applications is the phytase enzyme. This enzyme is a phosphohydrolase, which dissolves phosphate from phytate. Phytases are mainly used in monogastric animal feeding to improve phosphorus nutrition, reduce phosphorus pollution of animal waste, and save the phosphate sources^12^. The most common phytase enzymes used in the industry are derived from *Aspergillus niger* (*A. niger*) *and Aspergillus ficuum* (*A. ficuum*), which are usually produced by recombinant DNA technology in different hosts^13^.

Due to the high efficiency and specificity, enzymes are usually preferred to their chemical alternatives in terms of total cost, quantity, quality of final products, and environmental protection. Cost reduction is one of the most important steps of enzymes’ commercial production. Currently, commercial enzymes are produced in the recombinant form using biotechnological procedures. There are different ways to reduce the cost of recombinant protein production including the use of different methods of genetic engineering to develop new optimized hosts and constructs in the upstream process of manufacturing and optimization of cell culture conditions and protein purification^14,15^. Plasma treatment can be considered as a new potential technique for the optimization of recombinant protein production during different steps of manufacturing or improving the protein properties after being manufactured.

In this study, the recombinant yeast *P. pastoris* was constructed and grown to produce phytase protein in a secretory manner and the effects of ACP on the growth and productivity of the bioreactor yeast cells was evaluated. Furthermore, using CD and fluoresce spectroscopy, the direct effects of plasma on the phytase protein’s activity and structure were investigated.

## Results

### OES

**Figure 1:**
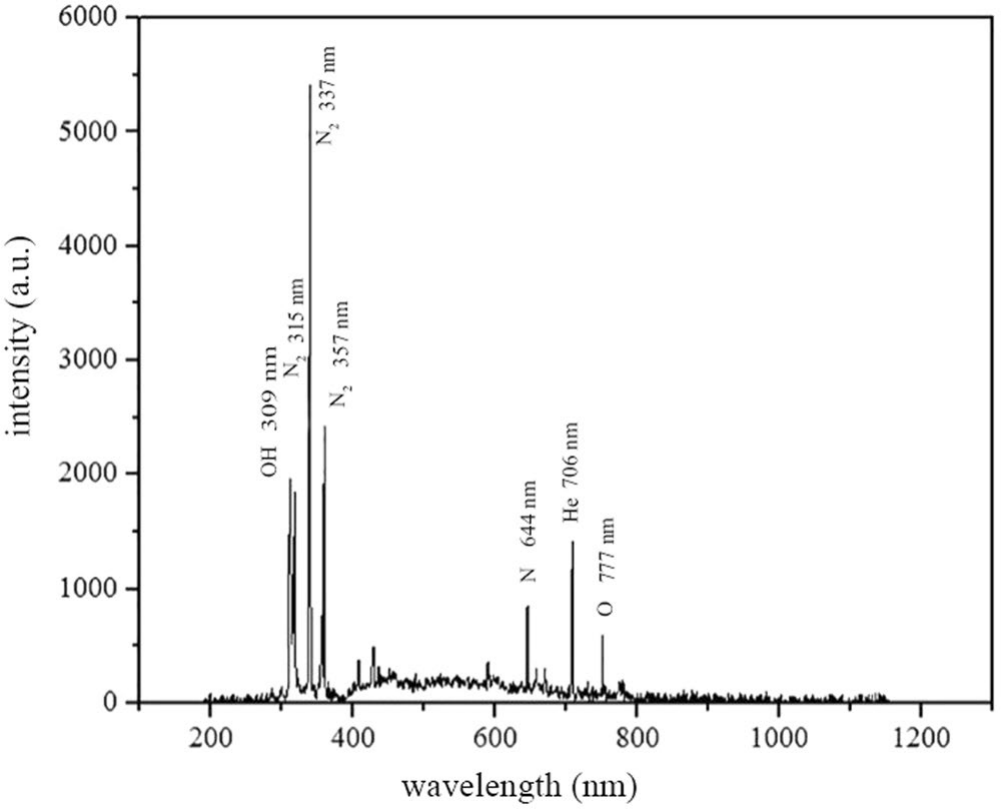
The emission spectrum of He plasma in air.

The emission spectrum of He plasma in air is indicated in Fig. 1. He plasma emission lines illustrate the presence of OH (309 nm), N_2_ (315 nm, 337 nm, 357 nm), He (706 nm), and O (777 nm)^16^.

### Construction of recombinant yeast and expression of phytase

**Figure 2:**
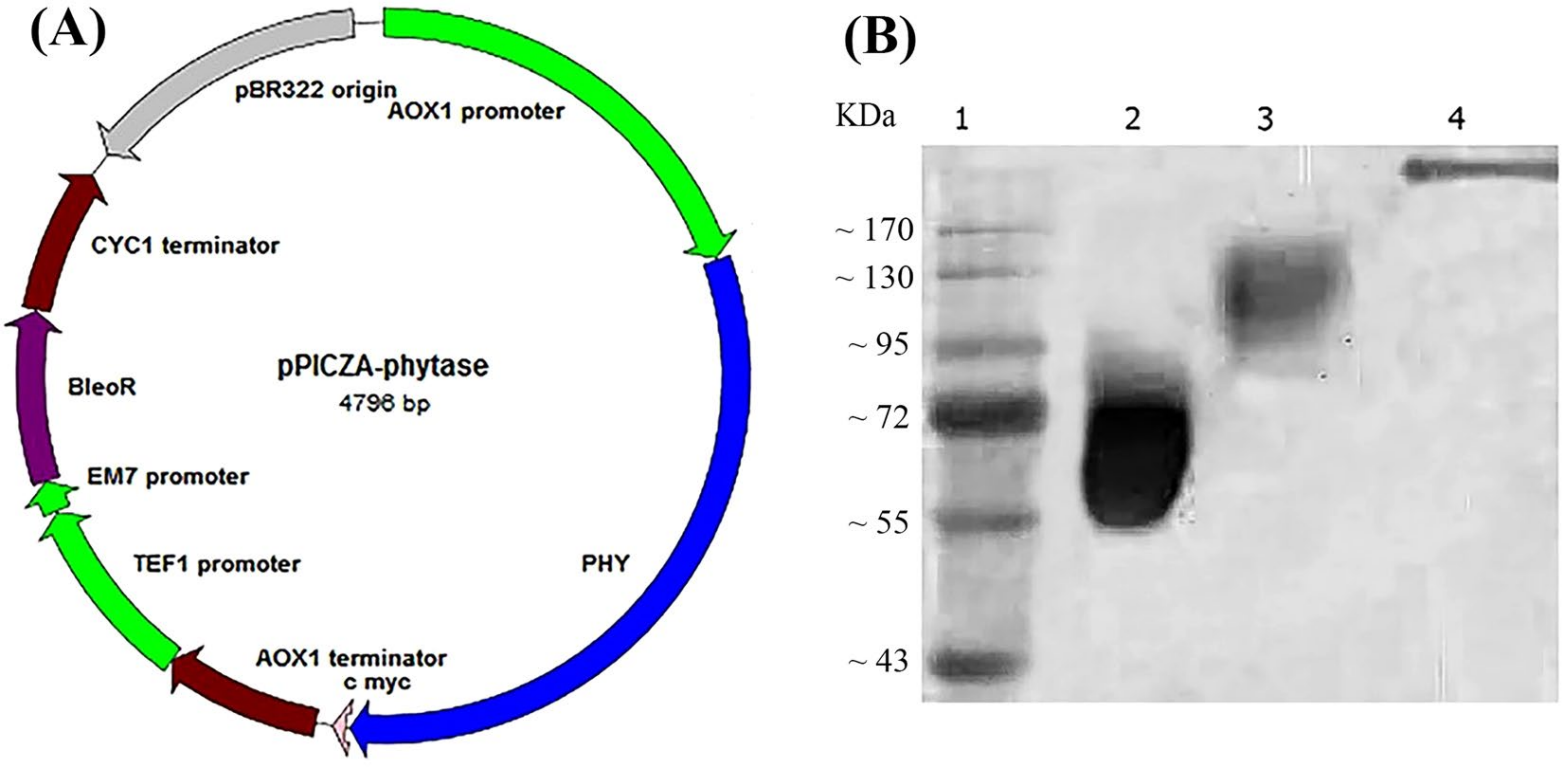
(**A**) The map of recombinant plasmid pPICZA-phytase. (**B**) SDS-PAGE of recombinant phytase produced in *P. pastoris*. Lane 1; protein marker, lane 2; commercial phytase, lane 3; recombinant phytase, lane 4; negative control (non-recombinant *P. pastoris*). Full-length gels are presented in Supplementary Fig. 1.

The correct construction of recombinant plasmid pPICZA-phytase (Fig. 2A) was confirmed by PCR, using phytase-specific primers, and sequencing. The transformant *P. pastoris* that incorporated the phytase gene, under the control of an AOX1 promoter, in its genome, was used for the expression of phytase in a secretory manner. After 96 h of induction with methanol, the supernatant of *P. pastoris* was subjected to SDS-PAGE (Fig. 2B). The results showed that *P. pastoris* efficiently produced phytase and secreted it using its native signal sequence. The molecular weight of recombinant phytase was more than 100 kDa, which was higher than the molecular weight of commercial phytase derived from *A. niger* and that of predicted from the *A. niger* phytase amino acid sequences (48.8 kDa).

### Effect of ACP on yeast cell growth

**Figure 3:**
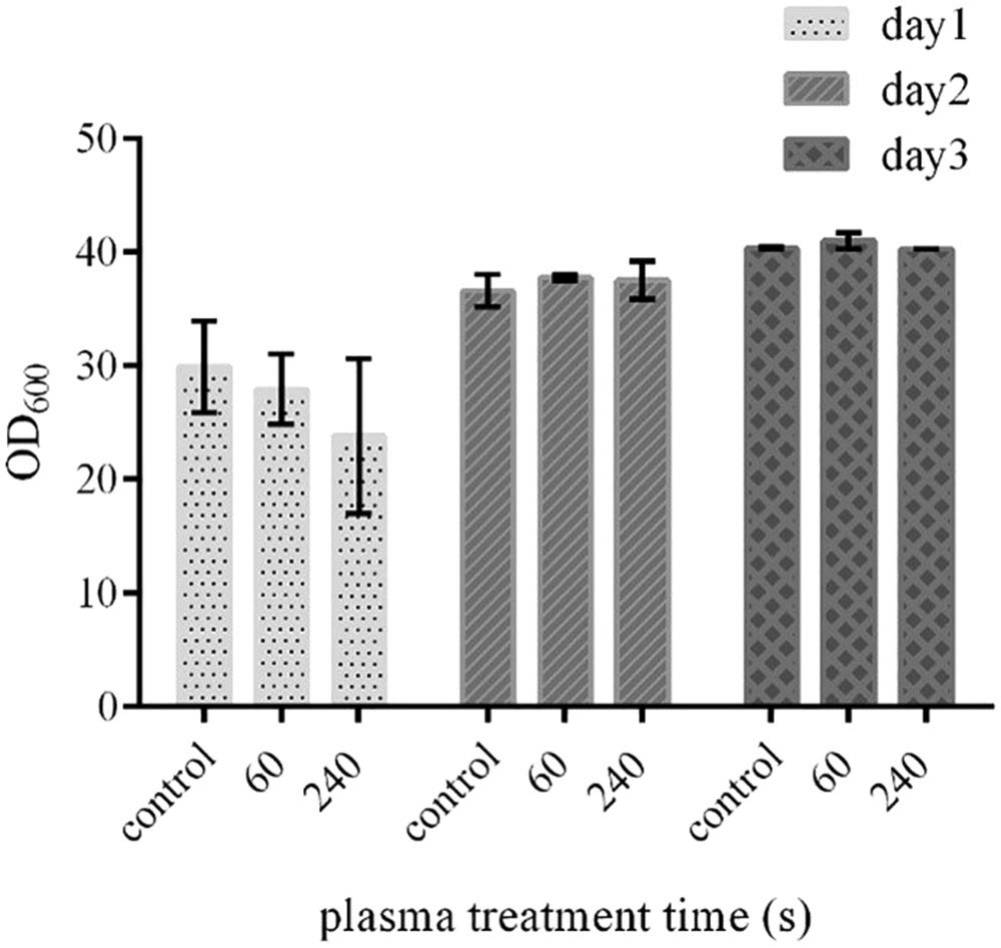
The effect of ACP treatment on the yeast cell growth. The negative effect of ACP on the yeast cell growth was observed on the first day, which was not significant and did not persist in the next two days. (*p ≤ 0.05; paired t-tests; N = 3). Error bars represent standard deviations.

The culture of yeast cells in ACP-treated culture medium led to a slight decrease in the growth in the first day, which was directly related to the duration of ACP exposure. However, this negative effect was diminished in the next two consecutive days and the measured yeast cell numbers did not show any significant difference between treated and untreated yeast cell cultures (Fig. 3).

### Effect of ACP on the recombinant protein production and activity

**Figure 4:**
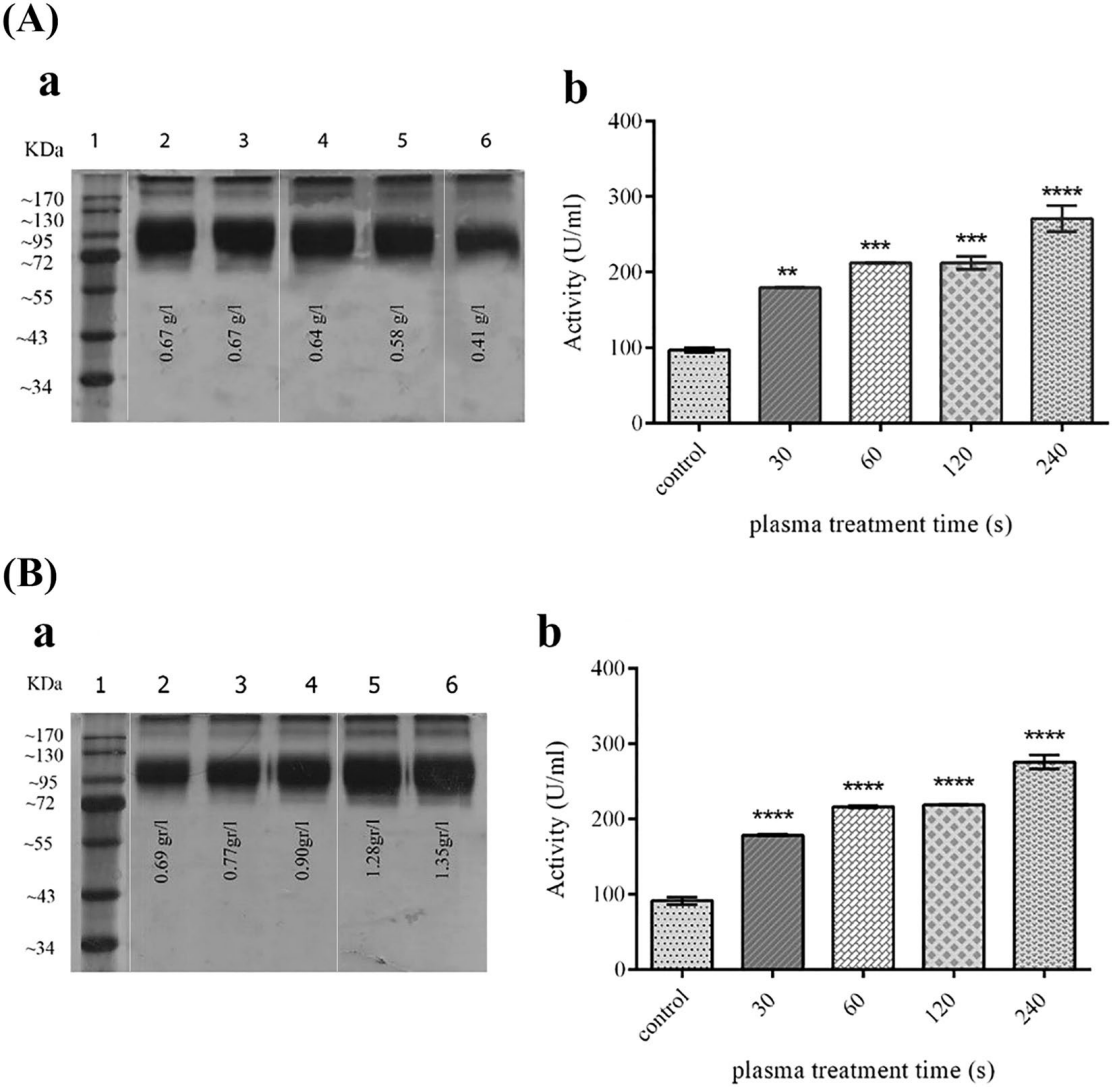
(**A**) The result of once treatment with ACP on recombinant phytase production and activity. (a) SDS-PAGE of recombinant phytase produced after once treatment of cell culture with ACP. Lane 1; protein marker, lane 2; control (without ACP treatment), lanes 3–6 belong to treatments with durations of 30, 60, 120, and 240 seconds, respectively. The amount of measured phytase is written on the gel figure. (b) The activity of recombinant phytase produced after once treatment of cell culture with ACP. (**B**) The result of multiple treatments with ACP on recombinant phytase production and activity. (a) SDS-PAGE of recombinant phytase produced after multiple treatments of cell culture with ACP. Lane 1; protein marker, lane 2; control (without ACP treatment), lanes 3–6 belong to every-day treatments with durations of 30, 60, 120 and 240 seconds, respectively. The amount of measured phytase is written on the gel figure. (b) The activity of recombinant phytase produced after multiple treatments of cell culture with ACP. (**p ≤ 0.01; ***p ≤ 0.001; ****p ≤ 0.0001; paired t-tests; N = 3). Error bars represent standard deviations. Full-length gels are presented in Supplementary Fig. 1.

The treatment of yeast cells with ACP led to a significant increase in enzyme activity, which was directly related to the ACP treatment duration (Fig. 4Ab). The repetition of ACP treatments in the next days of protein induction, which was applied directly to the culture containing the cells, did not lead to further increase in the measured enzymatic activity (Fig. 4Bb). While the results of SDS-PAGE showed a negative effect of the first ACP exposure on the recombinant protein productivity (Fig. 4Aa), the results of the ACP treatment repetition on yeast cells indicated contrary effects (Fig. 4Ba). The amounts of measured phytase protein (g/l) has been written on the gel figure.

### Effect of ACP on the phytase enzymatic activity

**Figure 5:**
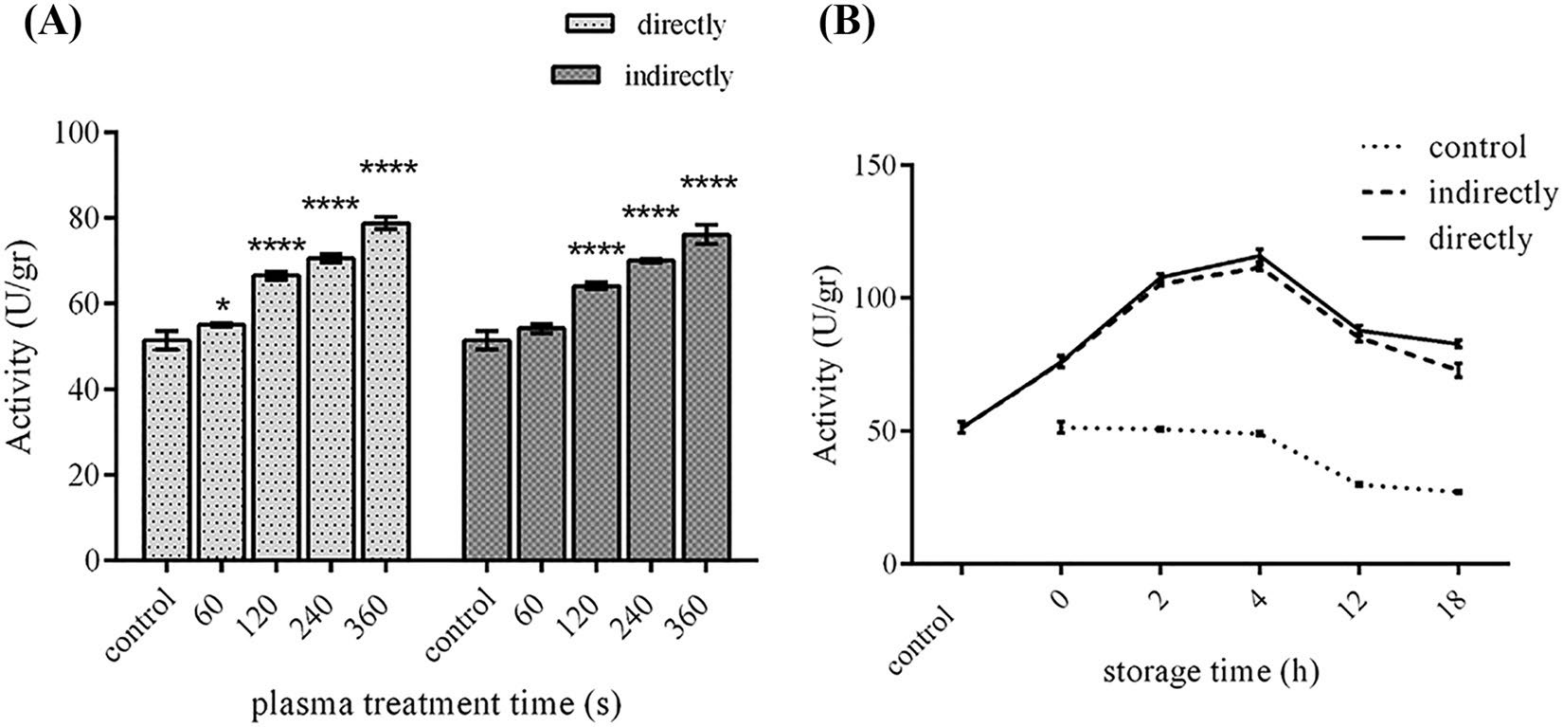
(**A**) The effect of direct and indirect ACP treatment time on the commercial phytase enzymatic activity. (**B**) The aging time of direct and indirect ACP treatment (360 seconds). (*p ≤ 0.05; ****p ≤ 0.0001; paired t-tests; N = 3). Error bars represent standard deviations.

Whether used directly to the enzyme solution or indirectly to the reaction buffer, ACP treatment caused a significant increase in the commercial phytase enzyme activity, which was directly related to the exposure time. 48% and 53% increase in the enzyme activity was observed for the maximum exposure time (360 seconds) (Fig. 5A). The persistence of ACP intensification effects on enzymatic activity (aging time) was investigated for 360 seconds of exposure and followed for 18 hours. As Fig. 5B indicates, the enzyme activity showed a sharp rise for the next 4 hours, resulting in 125% increase compared to the control. After that a decreasing trend began. Considering the control protein activity trend, this reduction was mainly related to the intrinsic protein half-life (Fig. 5B).

### Analysis of pH and temperature before and after ACP treatment

The measurement of the pH of the reaction mixture showed no difference in the effect of ACP treatment, while the temperature increased 8 °C after 6 minutes of exposure (from 8 to 16 °C).

### Effect of buffer pH on the ACP results on enzymatic activity

**Figure 6:**
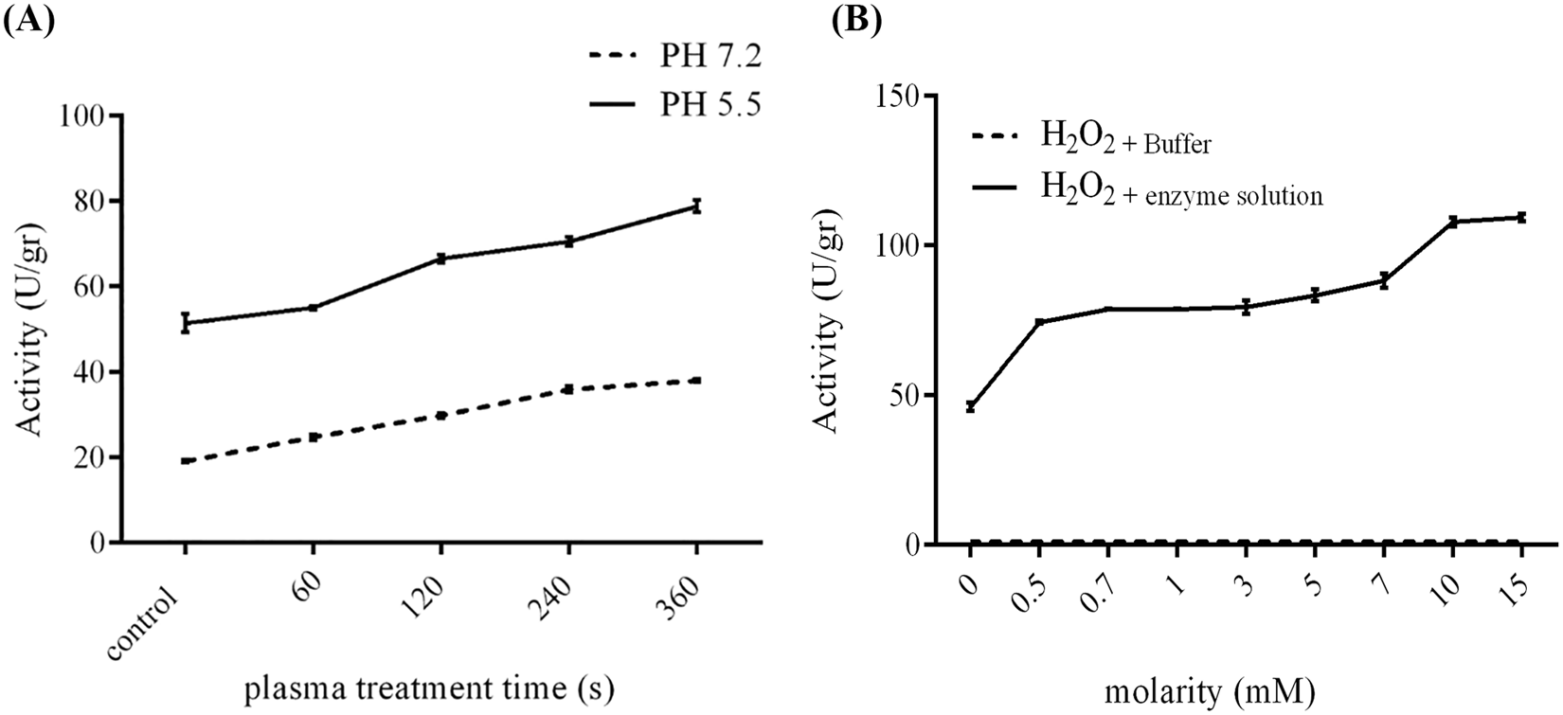
(**A**) The effect of pH on the ACP outcomes related to phytase activity. Increasing pH value leads to a decrease in phytase activity but with similar trends in ACP outcomes. (**B**) Effect of H_2_O_2_ on phytase enzymatic activity. The increase in the H_2_O_2_ concentration is associated with the improvement in enzymatic activity. (N = 3) Error bars represent standard deviations.

Increasing the pH of enzymatic reaction from 5.5 to 7.2 caused a significant decrease in the measured enzyme activity. The same trend was observed after the ACP treatment (Fig. 6A).

### The effects of H_2_O_2_ and temperature on enzymatic activity

Increasing concentrations of H_2_O_2_, up to 15 mM, improved the phytase activity. To ensure that H_2_O_2_ has no catalytic activity by itself, a negative control with no enzyme and the same concentration of H_2_O_2_ was used. The results indicated that H_2_O_2_ was not able to hydrolyze inorganic orthophosphate from phytic acid and the observed results were related to the effect of H_2_O_2_ on the phytase enzyme (Fig. 6B). Increasing temperature up to 40 °C indicated no effect on the enzymatic activity.

### H_2_O_2_ concentration after ACP treatment

**Figure 7:**
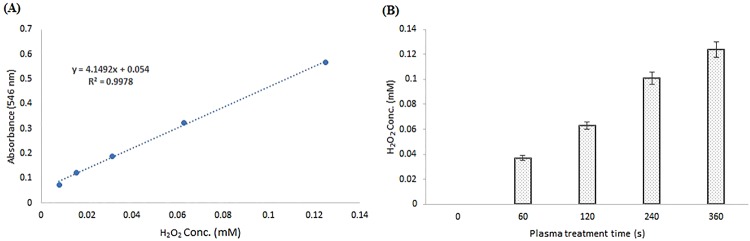
Measurement of produced H_2_O_2_ in sodium acetate buffer (pH 5.5) after ACP treatment using colorimetric assay. (**A**) The standard curve of H_2_O_2_ against the absorbance of 546 nm (**B**) Concentrations of produced H_2_O_2_ after exposure to ACP for 60–360 seconds.

The produced H_2_O_2_ was measured after ACP treatment of sodium acetate buffer (pH 5.5). The measured H_2_O_2_ in the treated buffer was amplified by increasing the treatment time and reached 0.125 mM after 6 minutes of treatment (Fig. 7A,B).

### CD analysis

**Figure 8:**
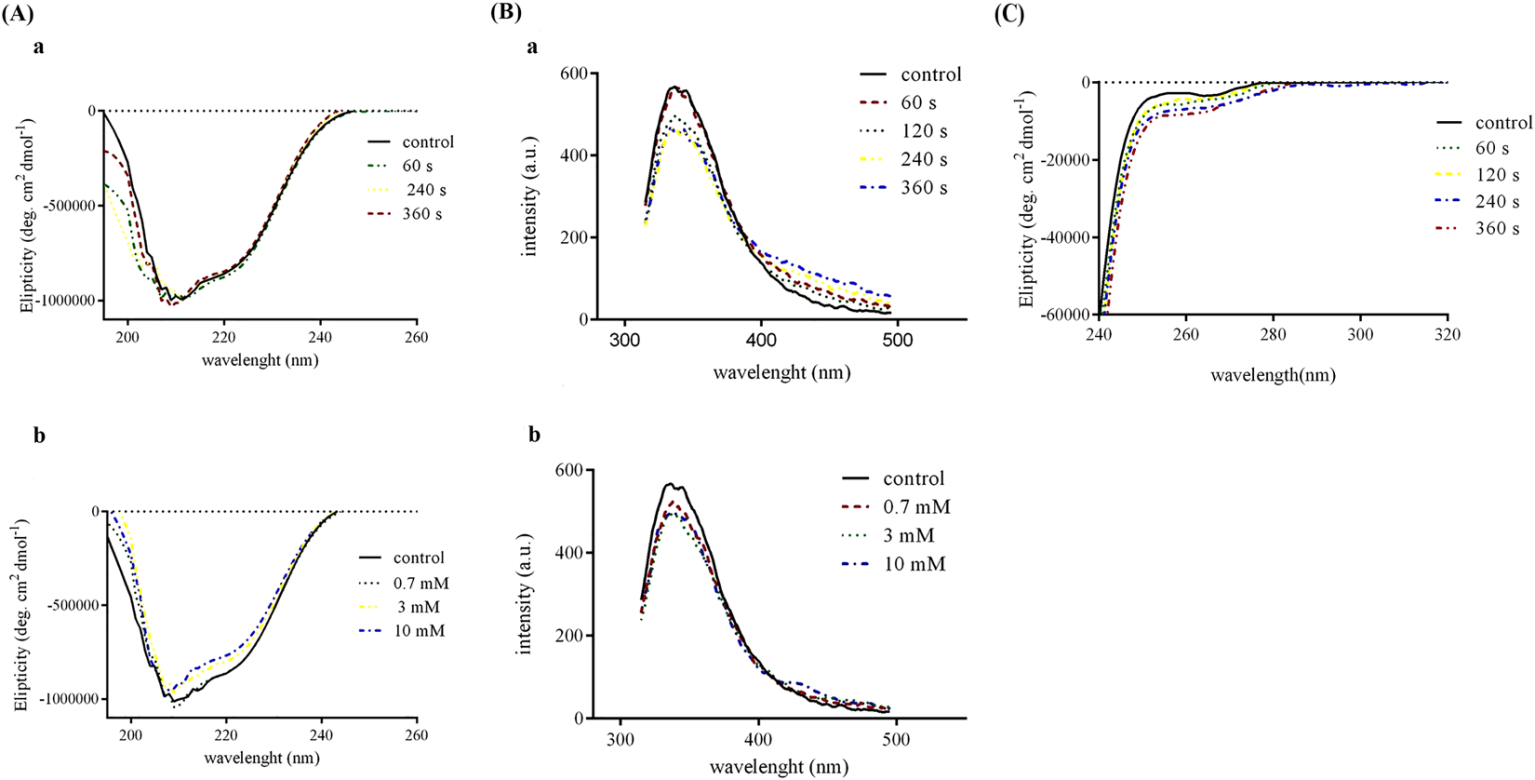
(**A**) Far-UV CD spectra of phytase protein solution. (a) After direct exposure to ACP for 60–360 seconds (b) After treatment with three different concentrations of H_2_O_2_. (**B**) Fluorescence analysis of phytase protein solution. (a) After direct exposure to ACP for 60–360 seconds (b) After treatment with three different concentrations of H_2_O_2_. (**C**) Near-UV CD spectra of phytase protein solution after direct exposure to ACP for 60–360 seconds.

The measurement of ellipticity is mostly used to follow the conformational changes of proteins caused by destabilizing factors. The results of far-UV CD indicated two typical negative peaks, 208 and 222 nm, corresponding to the percentage of α-helical structures. There was no significant change in the pattern of phytase molar ellipticity against wavelength for exposure times up to 6 min (Fig. 8Aa) and treatment with 0.7 mM H_2_O_2_. However, incubation with hydrogen peroxide at higher concentrations reduced the protein secondary structure (Fig. 8Ab).

The near-UV CD spectroscopy is mainly used for studying overall tertiary structure of proteins^17^. The obtained results indicated moderate changes in the CD spectrum of the protein after exposure to the ACP (Fig. 8C).

### Fluorescence analysis

The results of fluorescence analysis of phytase protein solution are shown in Fig. 8B. As can be inferred from the figure, there was a decrease in native fluorescence intensity upon increasing the plasma exposure time. The maximum emission spectrum was near 340 nm with an intensity of 560 a.u. Exposing to ACP seemed to have no observable effect on the fluorescence intensity during the first minute while increasing the exposure time decreased the intensity (Fig. 8Ba). As illustrated in Fig. 8Bb, the increase in the concentration of hydrogen peroxide is accompanied by a decrease in fluorescence intensity at 340 nm. The amount of reduction in maximum fluorescence intensity after 4 and 6 minutes of ACP treatment is somehow close to the results seen for 10 mM hydrogen peroxide treatment.

Furthermore, a difference in the fluorescence spectra of phytase exposed to ACP in the 400–500 nm region was observed, which probably could be attributed to the other forms of reactive species that existed in plasma.

### Phytase protein simulation

**Figure 9:**
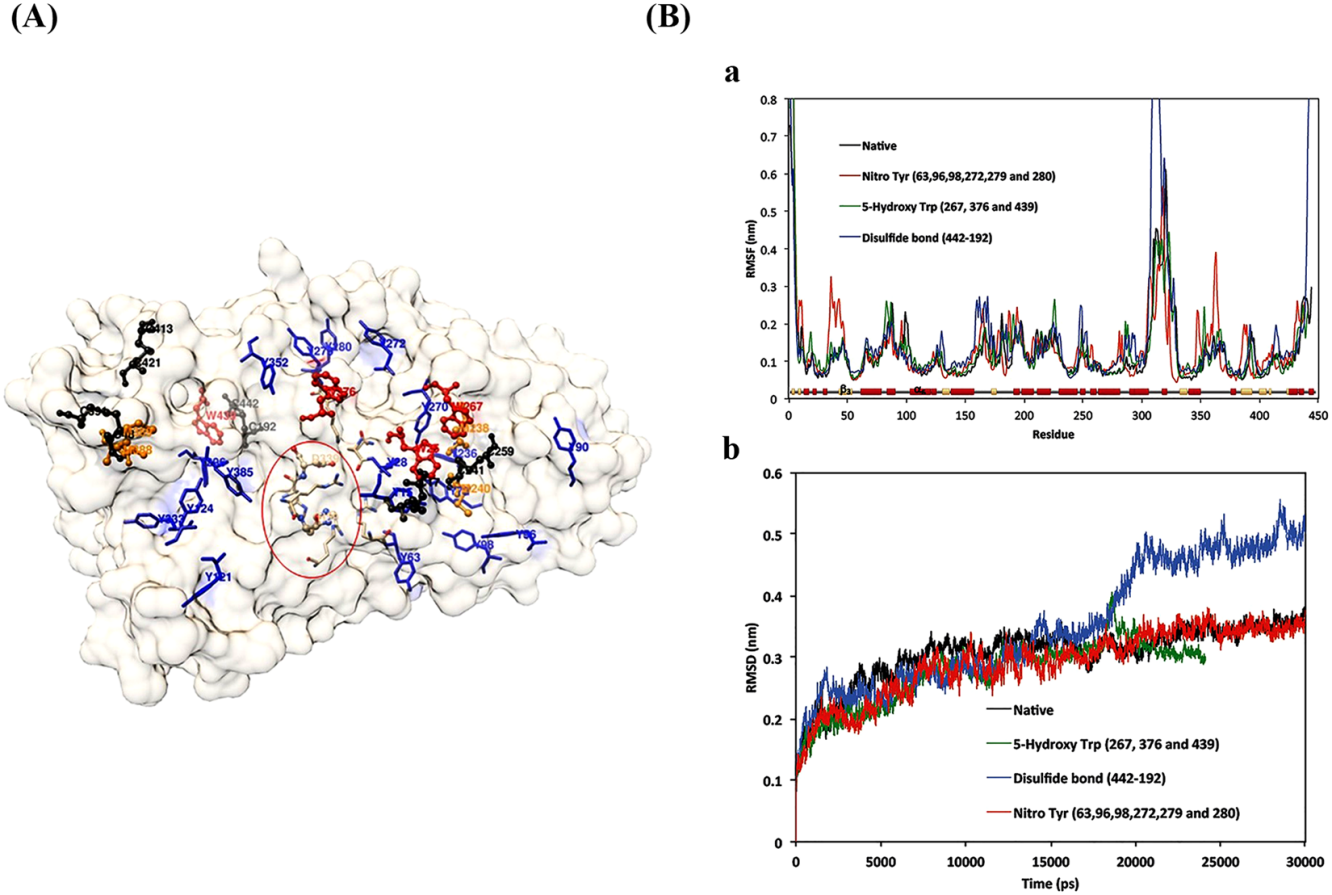
(**A**) Phytase protein simulation. The residues that are more sensitive to ROS and RNS are represented within the semitransparent surface of the protein. Met, Cys, Tyr, and Trp are colored in orange, black, blue, and red, respectively. The His and Asp residues in the catalytic site are colored in atom type and marked by a circle. (**B**) (a) RMSF per residue for native and modified phytase. The RMSF was calculated by best fitting the carbon alpha of the trajectory. Related secondary structures are assigned (b) RMSD during the simulation time for Native and modified structures.

As can be inferred from Fig. 9A, the residues susceptible to ROS/RNS and positioned closer to the surface (Cys, Met, Trp, and Tyr) might be affected initially. The His and Asp residues in the active site of the protein are displayed in the circle. According to the RMSF per residue for native and modified phytase, 3-nitro Tyr and 5-hydroxy Trp modifications had an inadequate effect only on the amino acid fluctuations, while disulfide bond breakage caused the loss of the secondary structure in the protein C-terminal (Fig. 9Ba). The RMSD against time graphs show similar patterns for 5-hydroxy Trp and 3-nitro Tyr residues. This is while the loss of the C- terminal disulfide bond led to an immediate increase in the RMSD, which was due to the loss of protein compactness (Fig. 9Bb).

## Discussion

In the present study, recombinant phytase was successfully produced in host *P. pastoris*. The results of SDS-PAGE indicated that the phytase protein from *A. niger* with the native signal peptide was secreted to the culture medium in high quantities and in the active form. The predicted molecular weight of this recombinant phytase was 48.8 kDa. However, the native phytase derived from *A. niger* has a higher molecular weight (about 65 kDa) and we obtained recombinant phytase with more than 100 kDa molecular weight. This higher molecular weight is suggested to be due to, at least to some extent, the presence of 10 sites of N-glycosylation in the phytase protein. In contrast to filamentous fungi such as *A. niger*, hyperglycosylation is a typical feature of yeasts, which most often synthesize large high-mannose type N-glycans and can result in molecular weight increases in glycoproteins like phytase^18^.

The reactive species produced by ACP could be either damaging or beneficial to biological systems depending on the time of exposure and plasma properties – i.e. voltage, gas type and the organism exposed. The effectiveness of ACP against different kinds of bacterial and fungal pathogens has been reported in multiple studies and this kind of plasma is presented in some novel anti-microbial technologies^19^. However, in this study, the non-lethal doses of He ACP were used due to its positive effects on the yeast cells productivity. To the best of our knowledge, this is the first time that the ACP is considered as a tool for the improvement of a host cell productivity. According to the results, the ACP was quite effective in increasing the measured recombinant enzyme activity and this incremental effect amplified by extending the time of exposure. While its enzymatic activity showed a significant increase, exposing the cultured cells in the medium to one dose of ACP slightly decreased the amount of recombinant phytase. On the other hand, exposing the cells with ACP in all subsequent days of culture led to increase in concentration of recombinant protein as well as phytase activity.

It was shown that free radicals and reactive species are linked to endoplasmic reticulum (ER) stress and the unfolded protein responses (UPR) in different cells, which activate a set of oxidative stress response genes, mainly those associated with protein folding. These responses mitigate the negative effects of ROS accumulation and hence increase the production capacity^20^. The plasma jet produces a variety of ions and free radicals in the gas phase. Free radicals and ions react with water to generate diverse biologically long and short lifetime reactive species in the liquid phase. Some of the long-lived species are as follows: hydrogen peroxide (H_2_O_2_), ozone (O_3_) and nitrate ion ($N{O}_{3}^{-}$); and short-lived species include hydroxyl radical (*OH*^−^), super oxide (${O}_{2}^{-}$), and singlet oxygen^21^. The contradictory effects of ACP on the recombinant protein production level for two different methods of exposure could be due to the fact that the repetition of cells treatment directly with ACP intensely turns on the stress response genes. Although this repetition of exposure increased the productivity, the enzymatic activity did not increase compared to the samples that were exposed just once. It seems that increasing the duration of ACP may not have more positive effects on the recombinant phytase activity.

Observing effects of ACP on the purified commercial phytase activity showed that using ACP in a direct or indirect manner leads to similar trends of increase in the phytase enzymatic activity. Slight differences observed could be due to the effect of short-lived reactive species on the produced protein in the direct mode. Following up the enzymatic activity indicated that the incremental effects of ACP continued for the next 4 hours and the posterior decline in the enzyme activity could be related to the intrinsic enzyme half-life, which was seen in the control. These results implied that most of the observed effects related to the phytase activity enhancement were due to the long-lived reactive species. Hao Zhang *et al*. found similar results when compared the direct and indirect effects of dielectric barrier discharge (DBD) plasma on lactate dehydrogenase enzyme^21^. However, in another study conducted by Li *et al*. no effect was observed in lipase activity when indirect exposure of He plasma jet was applied^9^. These contradictory observations could be due to the different plasma features or various physicochemical properties of proteins under study.

When studied separately, ultra-violate radiation and incubation in elevated temperatures, for short intervals (up to 6 minutes), did not have any effect on the phytase enzyme. This is while hydrogen peroxide caused an increase in the phytase activity, which is the reaffirming of the importance of long- lived species regarding the ACP effects.

The results indicated H_2_O_2_ and ACP treatment could affect the phytase secondary structure in different manners. According to CD analysis, increasing the dose of hydrogen peroxide caused small reductions in the phytase secondary structure, whereas ACP up to 6 minutes of exposure made no obvious change. This result may be attributed to the presence of five disulfide bonds in the phytase structure, which was preserved during the ACP treatment but affected by the direct treatment with H_2_O_2_. In addition, the measured quantity of produced H_2_O_2_ was significantly lower than the concentrations that affected the secondary structures (0.125 mM after 6 minutes of ACP treatment).

There was a reduction in the fluorescence intensity of the phytase enzyme, with no shift in the wavelength, after treatment with the ACP and H_2_O_2_. Furthermore, according to the results of near-UV CD (240–320 nm), slight alterations were detected in the tertiary structure of the phytase enzyme after exposure to the ACP. It seems that the ACP treatment moderately loosened up the tertiary structure of the phytase, which could be considered as an explanation for the observed fluorescence quenching. Previously, It has been shown that the plasma generated reactive species can cause modifications in biomolecules due to oxidation, nitration, dehydrogenation and dimerization of amino acids^22^. For instance, Takai *et al*. have showed that the quenching in lysozyme fluorescence intensity after exposure to APC was due to the modification of the Trp residues or Trp surroundings^23^. Altogether, the amino acid modifications and slight local changes in the tertiary structure around the modified amino acids can provide explanation for the observed effects of ACP on intrinsic fluorescence and near-UV CD spectra of the phytase.

Low concentrations of hydrogen peroxide had similar effects on enzyme fluorescence spectra as did the exposure to the ACP. However, the observed differences in the emission spectra might be attributed to the appearance of RNS in the case of ACP treatment.

A variety of free radicals can modify proteins. As a result, many different amino acid modifications can occur^24–26^. To compare the effects of ROS and RNS, low oxidative stress was simulated by modifying the accessible Trp residues, and for RNS, the tyrosine nitrosylation was considered. A high concentration of oxidative stress was also modeled by C-terminal (Cys, 413–421) disulfide bond breakage. The structural rigidity of *A. niger* phytase depends on the presence of 5 disulfide bonds (Cys residues; 8–17, 48–391, 192–442, 241–259, and 413–421). Therefore, while 5-Hydroxy Trp and 3-Nitro Tyr modifications did not affect the backbone RMSD, the breakage of the disulfide bond in presence of high concentrations of hydrogen peroxide showed a decrease in protein compactness compared to the native structure. The C-terminal disulfide bond breakage upon oxidation might have fewer effects compared to the breakage of other disulfide bonds in the structure.

Although phytase indicated better activity in an acidic buffer, it was found that a change in pH had no effect on the ACP treatment outcomes.

Finally, it is concluded that ACP in non-lethal doses can be considered as a new tool for the improvement of recombinant protein production in terms of productivity, and in the case of phytase enzyme, activity. In this study, it seemed ACP slightly decreased the compactness of phytase structure and exerted positive effects on its activity.

## Materials and Methods

### Strains, vectors, media and reagents

Yeast strain, the vector, and antibiotics were supplied from Invitrogen (USA). Phytic acid and sodium salt were purchased from Sigma-Aldrich (USA). Sodium acetate, sodium carbonate, sodium thiosulphate, ammonium heptamolybdate tetrahydrate, sulfuric acid 95–98%, glacial acetic acid, acetone, and glycine were supplied from Merck-Millipore (Germany). The plasmid DNA extraction kit, yeast genomic DNA extraction kit, and RNA extraction kit were obtained from SinaClon (Iran). Restriction enzymes were purchased from Thermo Scientific (USA). Commercial phytase was from Novozyme (Denmark).

### Preparation of pPICZA-phytase plasmid

The DNA coding sequence of phytase from *A. niger* was optimized according to the codon preference of *P. pastoris*. For cloning this modified ORF, two restriction recognition sites, *EcoR*I and *Kpn*I, were added to the 3′ and 5′ ends of the gene, respectively. The synthesized DNA fragment was inserted into a pPICZA plasmid using the aforementioned restriction enzymes and introduced into *E*. *coli DH5α* utilizing the heat shock transformation technique described by Sambrook and Russel^27^. The positive transformed bacterial clones were detected on low salt LB-agar plate (1% (w/v) tryptone, 0.5% (w/v) yeast extract, 0.5% (w/v) NaCl, 2% (w/v) agar, pH 7.0) containing 25 µg/ml of the antibiotic zeocin. The proper construction of recombinant plasmid was confirmed by PCR with specific phytase primers and DNA sequencing.

### Construction of recombinant Pichia pastoris expressing A. niger phytase

*P. pastoris* strain X-33 was used as a host for the expression of *A. niger* phytase. For this recombinant host to be prepared, wild-type X-33 was transformed with linearized pPICZA-phytase by electroporation (Gene Pulser, Bio-Rad, USA) according to the Easy Select™ *Pichia* Expression Kit instructions (Invitrogen, USA). The secretion of the gene product was directed by the native leader sequence of phytase and its expression was controlled by the AOX1 promoter during methanol induction. The recombinant yeast was detected on YPDS plates (1% (w/v) yeast extract, 2% (w/v) peptone, 2% (w/v) glucose, 18.2% (w/v) sorbitol, and 2% (w/v) agar) containing 100 µg/ml of the antibiotic zeocin. Recombinant *P. pastoris* strain X-33 expressing *A. niger* phytase was maintained as frozen stocks at −80 °C in 20% (v/v) glycerol.

### Expression of recombinant phytase

A small amount of the positive clones was placed on the YPDS agar plate and incubated at 30 °C for two days until colonies appeared and grew up to 1 to 2 mm in diameter. Some of these clones were inoculated into 5 ml of the yeast extract in YPG broth (1% (w/v) yeast extract, 2% (w/v) peptone, and 2% (v/v) glycerol) and cultivated at 30 °C with shaking at 250 rpm for 2 days. 1 ml of this culture was used to inoculate 50 ml of YPG. After two days, the medium was replaced with 50 ml YP containing 0.5% methanol to induce the expression of the phytase gene. The methanol feeding continued for three extra days with 1% concentration and shaking at 250 rpm, 30 °C. The culture growth was determined by OD_600_, the secreted phytase was analyzed on the last day with SDS-PAGE, and enzymatic activity was measured. The concentration of recombinant phytase was measured using the TotalLab software. The non-recombinant yeast *P. pastoris* was used as the negative control.

### Phytase enzymatic assay

The activity of phytase was determined using a colorimetric determination method introduced by Heinonen and Lahti with some modifications^28^. Briefly, 20 mM phytic acid solution in 200 mM sodium acetate buffer (pH 5.5) was used as the substrate. The reaction mixture containing 50 µl of enzyme solution was dissolved in 450 µl of buffer solution and incubated with 500 µl of substrate for 30 min at 37 °C. 2 ml of AMS color reagent solution (0.3% (w/v) ammonium heptamolybdate-tetrahydrate, 50% (v/v) acetone, and 25% (v/v) 5 N sulfuric acid 95%) was added to the mixture. 100 µl of acetic acid was added immediately to the solution to stop the reaction and the absorbance was measured using the spectrophotometer at a wavelength of 380 nm. The enzyme-free reaction mixture was used as the negative control. A standard curve was generated based on the serial dilutions of 10 mM KH_2_PO_4_ and used to quantify phytase activity equivalents. One unit of phytase was defined as the quantity of enzyme required to release 1 μmol of inorganic phosphate (P_i_) min^−1^ ml^−1^ or mg^−1^ under the assay conditions according to the following equation:

1 $$U=\frac{x\times n}{m\times t}$$

where *x* is the P_i_ concentration (μmol/ml), *n* is the dilution of the enzyme, *m* is the quantity of enzyme (mg or ml), and *t* is the time of reaction incubation.

### Atmospheric Pressure Plasma Jet (APPJ)

The plasma jet consisted of electrodes, dielectrics, and pulsed-dc power supply. A Pyrex tube (ID: 4 mm, OD: 6 mm) acted as the dielectric wrapped with a wire as a power electrode. The power electrode distance from the tube end was 10 mm. The plasma was run by a 10 kHz pulsed DC high voltage and voltage-variable (0–15 KV) power supply. The feeding gases were 99.999% pure helium (He) with a 2 l/min of gas flow rate.

### Optical Emission Spectroscopy (OES)

Plasma spectroscopy was employed for studying the plasma discharge characteristics. An ocean optic HR 2000 spectrometer was used to record optical emission and subsequently determine plasma essential species. The spectral range for this purpose was chosen from 200 to 11000 nm with an optical resolution of 0.5 nm. The spectra were subtracted from the baseline.

### Study of the effect of ACP on the yeast cell growth and recombinant protein production

ACP was applied to 20 ml of culture medium (YPG) in the Erlenmeyer, before addition of the yeast cells, for two time intervals of 60 and 240 seconds. The yeast cells were inoculated immediately to the ACP treated medium and the cell growth was followed for three consecutive days by measuring OD_600_.

The effect of ACP and its treatment duration on the recombinant protein production was determined using two procedures. In the first procedure the inductive media, containing 0.5% methanol, were exposed to the ACP for four different time intervals (30, 60, 120, and 240 seconds) and the equal number of cells, grown in the YPG, were inoculated to the treated media. 1% methanol feeding continued for three more days. In the second strategy, in addition to the first treatment of the empty inductive media with ACP, the culture media, containing the cells, were exposed to the ACP in the next days of methanol feeding (with the same duration).

### Effect of ACP on enzyme activity

The effects of ACP were investigated on recombinant and commercial phytase solutions. For this purpose, commercial *A. niger* phytase enzyme was prepared at 0.1 g/ml concentration in sodium acetate buffer (0.2 M, pH 5.5).

**Figure 10:**
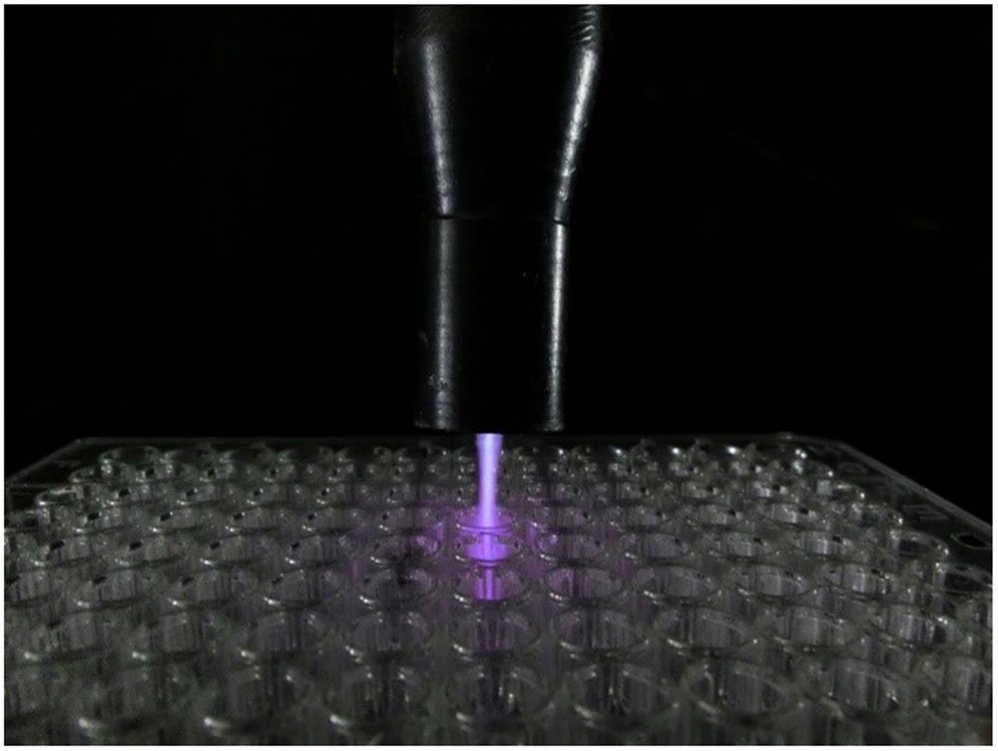
Helium plasma jet applied at the distance of 1 cm from the surface of 96-well plate.

ACP treatments were performed directly by exposing the plasma to the reaction buffer immediately before enzyme addition and indirectly by exposing the plasma to the enzyme solution. 300 µl of enzyme solutions were loaded in a 96-well plate and the plasma was applied at the distance of 1 cm from the well surface (Fig. 10) at four different durations (1, 2, 4, and 6 minutes). The untreated enzyme solution was used as the negative control. To avoid the temperature fluctuation, the enzyme solution was kept at 4 °C during the ACP treatment. The enzymatic activity was measured according to Equation 1.

### Effect of plasma on the pH and temperature

To study the effect of plasma on the pH and temperature of the exposed medium, these two parameters were measured before and after 6 minutes of plasma treatment in the commercial phytase enzyme solution (0.1 g/ml concentration in sodium acetate buffer, pH 5.5).

### Study of the effect of buffer pH on the ACP results

Based on our analysis (data not shown), pH 5.5 was optimum for commercial *A.niger* activity, while at pH 7.2, up to 60% of the enzyme activity is lost. To evaluate the effects of enzyme solution pH on the obtained results from the ACP treatment, the commercial enzyme was dissolved in the PBS buffer (pH 7.2) and exposed to the ACP with the same duration. The results were compared against the previous assays in sodium acetate buffer (0.2 M, pH 5.5).

### Effect of H_2_O_2_ and temperature on enzyme activity

To find out which products of ACP (*e.g*. H_2_O_2_ or local increase of the temperature inside plasma) were corresponded to the obtained results, H_2_O_2_ and temperature were applied separately to the enzyme solutions. For this purpose, H_2_O_2_ from 0.2 mM to 15 mM concentration levels was subjected directly to the enzyme solution (0.1 g/ml). The hydrogen peroxide was prepared as in previous studies^29,30^. The effect of temperature was studied up to 40 °C.

### H_2_O_2_ concentration measurement

H_2_O_2_ concentration in the ACP treated buffer (sodium acetate, pH 5.5), with different treatment times, was measured using an H_2_O_2_ assay kit (ZellBio GmbH, Germany) following the manufacturer’s protocol. Produced H_2_O_2_ was determined based on the plotted standard curve.

### Fluorescence measurement

The fluorescent intensity of the phytase enzyme solution (2 mg/ml) in sodium acetate buffer (0.2 M, pH 5.5) was measured using a spectrophotometer (Carry Eclipse50, Germany). For this purpose, the excitation wavelength of 295 nm was used and the emission spectra were recorded at 310–500 nm.

### Circular dichroism spectroscopy

To evaluate the effect of ACP on the content of regular secondary structures, the far-UV CD (190–260 nm) and near-UV CD (240–320 nm) spectroscopy were employed using an Aviv model 215 spectropolarimeter (USA). Phytase solution was prepared in sodium acetate buffer (0.2 M, pH 5.5) at 6 mg/ml and 3 mg/ml concentration for far- and near-UV CD, respectively.

The solutions were treated by the ACP and incubated at room temperature for 30 min. The experiments were carried out using 0.1 cm and 1 cm path length quartz cuvette for far- and near-UV CD, respectively.

The results were represented as ellipticity (deg. cm2 dmol^−1^), which was determined using AVIV software^31^ according to the following equation:

2 $${\rm{Ellipticity}}[{\rm{\theta }}]=\frac{millidegrees\times mean\,residues\,weight}{pathlength\,in\,millimeters\,\times concentration\,in\,mg\,ml-1}$$

The data obtained for both untreated (control) and treated enzyme solution samples were obtained from three replicates. The results were subtracted from the buffer CD signal and smoothed.

### Molecular dynamics simulations

The MD simulations were based on the 2.2 Å crystal structure of *A. niger* phytase in the complex with myo-inositol hexasulfate (Protein Data Bank entry 3K4Q). For the molecular dynamic simulation, heterogeneous groups were deleted from the structure before further investigation. The missing residues and atoms were added using modeler. The parameterization of modified residues was performed according to Vienna-PTM and GROMOS54A8 force field^32^. According to the predicted modifications, which were created on the protein structure and surface amino acids due to the ACP treatment, three more modulations were performed in addition to natural phytase. Each of them contained one of the expected modifications. The 5-hydroxy Trp and cysteic acid were considered the products of ROS and 3-Nitro Tyr reflected the effect of RNS on the protein. The SPC water model and 0.01 M NaCl were used to solvate the native and modified structures. The NVT was performed for 30 ns using LINKS algorithm for bond parameters, Varlet cutoff-scheme, Particle Mesh Ewald electrostatics, and Berendsen thermostat (300 K, tau_t = 0.1). Following NPT, MD was performed for 30 ns and the energy and coordinates were recorded every 2500 steps. The trajectories were then analyzed using GROMACS tools. The root-mean-square deviation (RMSD) for backbone atoms and root mean square fluctuation (RMSF) per carbon alpha were compared in native and modified structures.

### Statistical analysis

All values are represented as the mean and standard deviation (SD) of four replicates. The significance values were calculated as compared to the control. Statistical significance between data points was determined using one-way ANOVA. Significant differences were judged at *p < 0.05 and ****p < 0.0001.

## Electronic supplementary material

Supplementary file
