# Supplementary material for: The effect of non-thermal atmospheric plasma on the production and activity of recombinant phytase enzyme
Source: Sci Rep. 2018 Nov 9;8:16647. doi: 10.1038/s41598-018-34239-4 (PMC6226467; doi:10.1038/s41598-018-34239-4)
Supplement: Supplementary file 1 — Supplementary file [file 41598_2018_34239_MOESM1_ESM.pdf]

# The effect of non-thermal atmospheric plasma on the production and activity of recombinant phytase enzyme

**Mahsa Farasat[1], Sareh Arjmand[2,*], Seyed Omid Ranaei Siadat[2], Yahya Sefidbakht[2], Hamid Ghomi[1]**

[1]Laser and Plasma research Institute, Shahid Beheshti University, G. C., Tehran, Iran

[2]Protein Research Center, Shahid Beheshti University, G. C., Tehran, Iran

*Correspondence: Sareh Arjmand, Protein Research Center, Shahid Beheshti University, G. C., Tehran, Iran, PO Box 1983969411, Tel +98(21)29905010, Fax +98(21)22434500 , Email s_arjmand@sbu.ac.ir

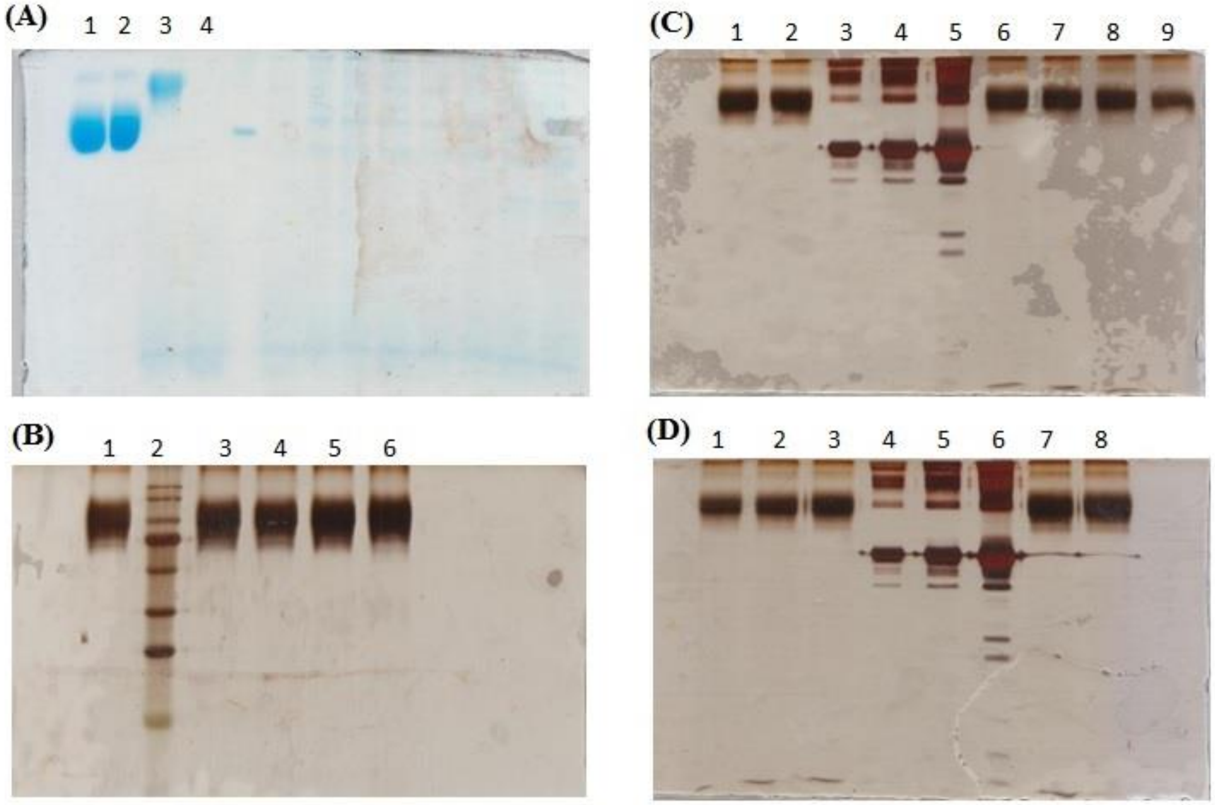


***Supplementary figure 1.*** *Full-length gels*
